# Perceiver and target partisanship shift facial trustworthiness effects on likability

**DOI:** 10.1038/s41598-023-33307-8

**Published:** 2023-04-15

**Authors:** Caraline S. Malloy, Colleen Hughes, Brittany S. Cassidy

**Affiliations:** 1 Baruch College, New York, USA; 2 Montreal Neurological Institute, Montreal, Canada; 3 Department of Psychology, University of North Carolina at Greensboro, 296 Eberhart, PO Box 26180, Greensboro, NC 27412 USA

**Keywords:** Human behaviour, Social evolution

## Abstract

The affective polarization characteristic of the United States’ political climate contributes to pervasive intergroup tension. This tension polarizes basic aspects of person perception, such as face impressions. For instance, face impressions are polarized by partisanship disclosure such that people form positive and negative impressions of, respectively, shared and opposing partisan faces. How partisanship interacts with other facial cues affecting impressions remains unclear. Building on work showing that facial trustworthiness, a core dimension of face perception, is especially salient for ingroup members, we reasoned that shared and opposing partisanship may also affect the *relation* between facial trustworthiness characteristics and subsequent likability impressions. A stronger positive relation emerged for shared versus opposing partisan faces across more conservative and liberal perceivers (Experiments 1 and 2). Exploratory analyses showed that this difference links to perceived partisan threat (Experiment 1) and that experimentally manipulating inter-party threat strengthened opposing partisan derogation and shared partisan enhancement patterns (Experiment 2). These findings suggest that partisanship extends from affecting overall face impressions of partisans to affecting the relation between a core dimension of face perception and subsequent impressions. These findings highlight the prevalence of partisanship effects in basic aspects of person perception and have implications for intergroup behavior.

## Introduction

Over the past several decades, Americans have become increasingly polarized with regard to their political partisanship^1,2^. Although scholars debate whether this polarization is reflected in policy preferences^3^, considering partisanship has become important to understand how people respond to others^4,5^. For example, behavior change interventions using descriptive norms are more powerful when norms are established by leaders with shared relative to opposing partisanship^6,7^. Such findings reflect affective polarization, in which people mistrust opposing partisans^8^. Beyond considering how partisanship relates to policy-related behaviors^9^, a growing body of work has examined whether partisanship polarizes basic judgments characteristic of person perception. For example, people rate the faces of others with opposing relative to shared partisanship as less attractive^10^, likable^11^, and competent^12^. Whether partisan information affects oft-studied relations between fundamental ways people perceive faces and such evaluations is unclear. This possibility is important because beyond people stating that they like faces based on partisan information, this information could *shift* how people integrate primary aspects of face perception into their impressions. Partisanship may thus affect how core dimensions of face perception^13^ contribute to face impressions. Here, we present two experiments characterizing perceiver and target partisanship effects on the well-established positive relation between facial trustworthiness and evaluated likability.

Facial trustworthiness is a core dimension of face perception conveying valence^13^. Impressions based on this dimension (e.g. likability) are spontaneous^14^ and persist over time^15,16^. Facial trustworthiness affects evaluations of targets’ personalities and intentions^17^, ultimately increasing the likelihood of approaching targets who have higher facial trustworthiness^18^. These faces are more likable to perceivers, which reflects that increasing facial trustworthiness broadly correlates with more positive judgments^13^. It is important to note that although facial trustworthiness and evaluated likability are related, they are still distinct from each other. Indeed, computational models of facial likability related more to attractiveness than to trustworthiness and competence^19^. Examining the relation between facial trustworthiness and evaluated likability thus does not simply reflect the same construct measured through database norms and by participant-given evaluations.

People rely on facial trustworthiness when making decisions^20,21^. For example, people playing economic games are more likely to interact with partners with high facial trustworthiness^22,23^, perhaps because likability signals a willingness to cooperate^24^. Despite the gravity of consequences relating to facial trustworthiness^25^, facial trustworthiness does not reliably indicate underlying traits^26,27^. Thus, reliance on facial trustworthiness may lead people to have unfair likability evaluations that perpetuate interpersonal tension. Identifying factors that *shift* facial trustworthiness effects on subsequent evaluations is especially relevant in the political domain, where interpersonal tension prohibits meaningful discourse^1^.

Many studies on the relation between facial trustworthiness and evaluations like likability have been conducted without other information^13^. Faces often do not exist in the absence of other information, however, allowing for the possibility that external information may shift face impressions. Supporting this possibility, faces belonging to racial groups stereotyped as untrustworthy are seen as more untrustworthy than faces of group members not subjected to this stereotype^28,29^. Even simply labeling faces affects impressions. For example, labeling faces as having non-visible illnesses relative to being healthy elicits more negative impressions of them^30^. In this work, faces were held constant across conditions, meaning that only external information affected impressions. Complementary findings have emerged in the political domain. Partisans, for instance, rate faces as less attractive if they are labeled as having dissimilar candidate preferences^10^. Partisans also shift their impressions to be more positive and negative based on whether targets have shared relative to opposing partisanship^11,12^.

When considering their own groups, people are especially sensitive to high facial trustworthiness. Across five experiments, faces high on facial trustworthiness conveyed powerful positive information causing people to include more highly trustworthy faces in their ingroups^31^. This pattern emerged independent of target race and sex, suggesting that high facial trustworthiness can outweigh category cues when people determine with whom they will affiliate. Further, this pattern aligns with work showing that people exclude others believed to be “bad” for the ingroup^32,33^. It also reflects a facial trustworthiness effect on ingroup over-exclusion, whereby people reserve group inclusion to those who may provide a group benefit^34^. Overall, these findings suggest reliance on a core dimension of face perception associated with subsequently assumed personality traits^13^ and intentions^22^ over at least some group information when including others with their groups.

That people determine their own partisanship, however, means that faces with a range trustworthiness are shared or opposing partisans to perceivers. That high facial trustworthiness is weighed heavily when considering ingroup membership^31^ raises the possibility that external information, like partisanship cues, may *differentially* affect impressions based on facial trustworthiness. Here, we test whether partisanship alters the relation between facial trustworthiness and likability. Such a pattern would be consistent with work showing a larger investment advantage for trustworthy over untrustworthy faces when faces are of ingroup (versus outgroup) members^35^ and link to work showing that context enhances evaluations based on facial trustworthiness for some faces over others^36^. It would also identify that facial trustworthiness relates to evaluations of group members determined by self-selection, thus extending the literature on the interplay between core dimensions of face perception and group membership. Examining the nature of these patterns is also of interest. One possibility was that people would evaluate shared relative to opposing partisans as more likable overall. Complicating this overall pattern, it could also be that partisanship will affect the relation between facial trustworthiness and likability such that evaluations will also be stronger for shared relative to opposing partisans. Another possibility was that the strong relation between facial trustworthiness and likability would elicit shared relative to opposing partisans being evaluated as more likable *only* when they have higher facial trustworthiness. Given lower trustworthiness, opposing relative to shared partisans may be evaluated more positively if people treat the latter as “black sheep” to maintain a positive social identity^37^.

Regardless of their nature, these patterns may provide a novel way to understand partisan tension. If the positive relation between facial trustworthiness and evaluated likability is stronger for some partisans than others, it would suggest that facial trustworthiness may provide context-specific benefits. For example, placing more value on higher trustworthiness of shared relative to opposing partisan faces could perpetuate tension by limiting opportunities for the contact that reduces affective polarization^38^.

Across two experiments, we show the positive relation between facial trustworthiness and evaluated likability to be stronger for shared relative to opposing partisans. For the benefit of future work, we test *why* this shift emerges on an exploratory basis. Negative impressions of opposing partisans relate to the threat they are perceived to pose^39^, a pattern consistent with politically relevant stimuli affecting attitudes based on their elicited threat^40^ concomitant with inter-party competition^41^. Notably, perceived partisan threat exacerbates impressions of other partisans^12^. We thus reasoned that more threatening contexts may explain the extent to which this relation is stronger for shared partisans relative to others. We provide evidence for this possibility through self-reported perceived partisan threat (Experiment 1) and an experimental manipulation (Experiment 2).

## Experiment 1

Experiment 1 tested whether the positive relation between facial trustworthiness and evaluated likability is stronger for shared relative to opposing partisans. People saw faces varying in facial trustworthiness one at a time and evaluated their likability. In addition to faces being labeled as Republicans or Democrats, a subset had no partisan information. Including unlabeled faces allowed for a more nuanced interpretation of the expected pattern. Because people over-exclude others from their ingroups^34^, facial trustworthiness should strongly relate to evaluated likability among shared partisans. If over-exclusion broadly devalues the high facial trustworthiness of targets without shared partisanship, this positive relation should be stronger for shared partisan faces relative to both unlabeled and opposing partisan faces. Because opposing partisans are perceived more negatively than independent individuals^5^, another possibility was that a stronger relation would emerge for unlabeled relative to opposing partisan faces because the latter reflects an especially derogated group^1^. Because partisan animus is linked to perceived threat^39^, we examined whether the intergroup effect was associated with threat on an exploratory basis. Specifically, we explored whether perceived partisan threat related to the extent to which the positive relation was stronger for shared relative to opposing partisans.

## Method

### Participants

Experiment 1 comprised a design assessing interactive effects of partisan label (Republican, Democrat, unlabeled), facial trustworthiness (continuously measured), and perceiver political ideology (continuously measured). Because we did not have frame of reference to predict the effect size of the hypothesized three-way interaction, we targeted a 125-person sample based on recent work showing that people over-include trustworthy faces in their ingroups^31^. We oversampled to account for expected manipulation check failures and to increase the likelihood of obtaining a wide range of political ideologies. Overall, 150 people (*M*_*age*_ = 38.79, *SD* = 11.45; *M*_*years of education*_ = 15.60, *SD* = 3.63; 56 female) from the United States were recruited from Amazon’s Mechanical Turk (MTurk). Of these people, 131 reported identified as White, nine as Black, six as Asian, and three as multi-racial. One person’s race was unreported. Of these people, 140 identified as non-Hispanic. The University of North Carolina at Greensboro IRB approved all experiments. All experiments were performed in accordance with relevant guidelines and regulations. All participants provided informed consent.

### Task

**Figure 1:**
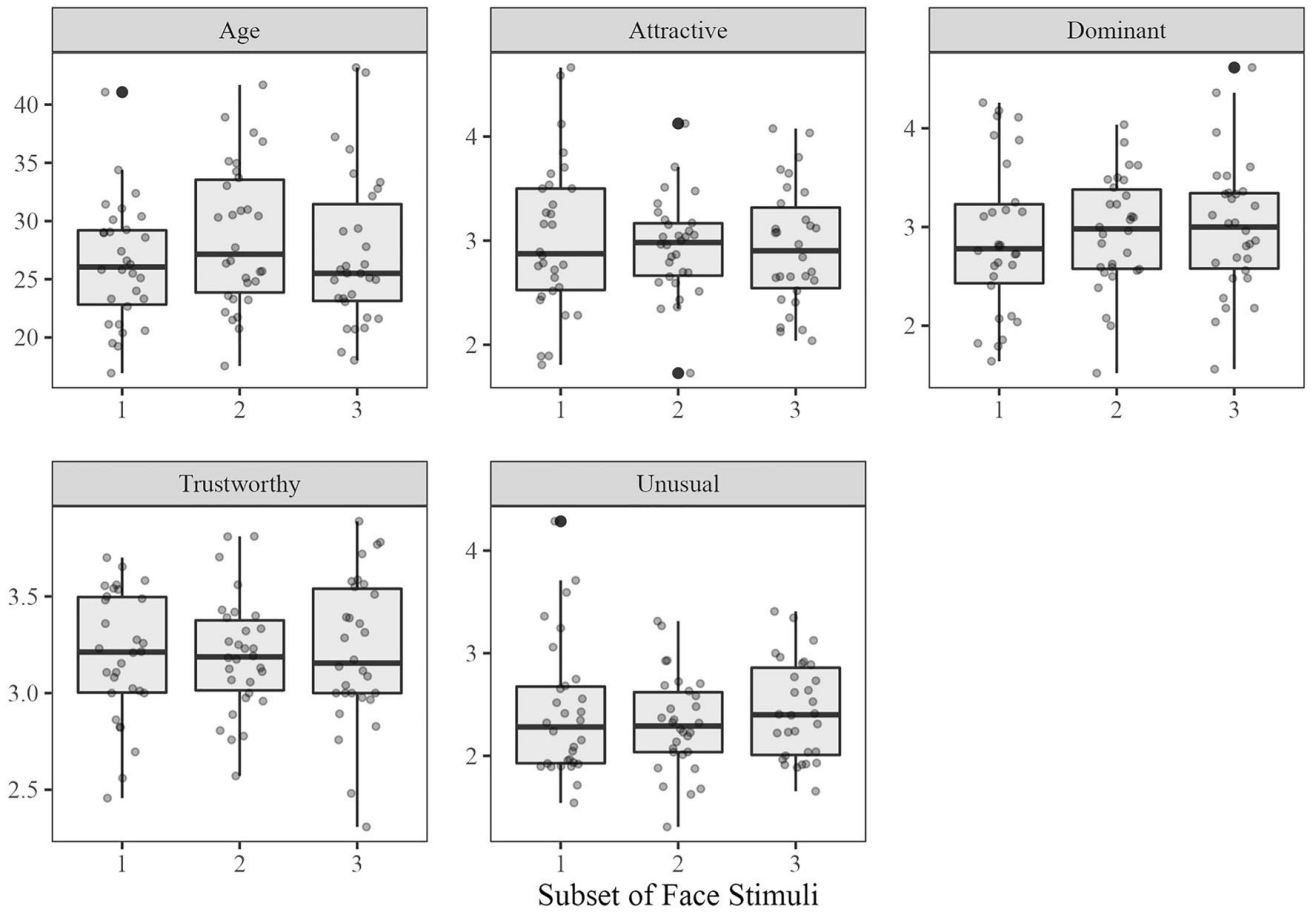
Norming data for the face subsets in Experiments 1 and 2^42^. Trait ratings were given on a 7-point scale (1 = not at all, 7 = extremely).

Ninety neutrally expressive adult White male faces were drawn from the Chicago Face Database^42^. Thirty were randomly paired with each of three labels (Republican, Democrat, or unlabeled). Three versions counterbalanced face-label pairings (e.g. faces labeled as Republican in one version were labeled as Democrat in a second and were unlabeled in a third). Five ANOVAs showed the face sets had similar age, attractiveness, dominance, trustworthiness, and unusualness, all *F*s < 1.13, all *p*s > 0.33. See Fig. 1 for value ranges. Here, facial trustworthiness (range: 2.31–3.89 on a 7-point scale of increasing trustworthiness), as a predictor of evaluated likability, was derived from these norms. See OSF for a list of the selected faces.

Participants completed a self-paced randomized 90-trial task in which they evaluated face likability on a scale ranging from 1 (*not at all likable*) to 7 (*extremely likable*). They saw one face per trial. They were told that some faces would have partisan labels (Republican or Democrat), others would not, and that they could use the information as they saw fit. After the task, they indicated adherence (“Did you randomly select your ratings for faces?”) on a scale from 1 (*not at all*) to 5 (*completely*) (*M* = 1.16, *SD* = 0.60) and whether they rated faces or cars. All indicated faces.

### Partisanship characterization

We collected partisanship characterization measures in a random order after the task. See Table 1 for descriptives and intercorrelations.

**Table 1:** Intercorrelations between partisanship characterization measures in experiment 1**.**

Variable	*M*	*SD*	1	2	3	4	5	6
1. Political ideology	4.72	1.69						
2. Perceived threat (Republican)	4.05	1.91	0.64** [0.54, 0.73]					
3. Perceived threat (no party)	2.47	1.21	− 0.10 [− 0.26, 0.06]	0.12 [− 0.04, 0.28]				
4. Perceived threat (democrat)	3.07	1.89	− 0.49** [− 0.60, − 0.35]	− 0.09 [− 0.25, 0.07]	0.42** [0.28, 0.54]			
5. Affiliation (Republican)	2.45	2.04	− 0.74** [− 0.80, − 0.66]	− 0.57** [− 0.67, − 0.46]	0.25** [0.09, 0.39]	0.48** [0.35, 0.59]		
6. Affiliation (no party)	3.27	2.17	− 0.09 [− 0.25, 0.07]	0.06 [− 0.10, 0.22]	− 0.11 [− 0.26, 0.05]	0.15 [− 0.01, 0.31]	− 0.07 [− 0.23, 0.09]	
7. Affiliation (democrat)	4.01	2.27	0.73** [0.65, 0.80]	0.48** [0.34, 0.59]	− 0.03 [− 0.19, 0.13]	− 0.57** [− 0.67, − 0.45]	− 0.48** [− 0.59, − 0.35]	− 0.31** [− 0.45, − 0.16]

*M* and *SD* are used to represent mean and standard deviation, respectively.

Values in square brackets indicate the 95% confidence interval for each correlation.

* indicates *p* < .05.

** indicates *p* < .01.

#### Perceiver political ideology

Participants indicated political ideology over four items (overall, social issues, economic issues, and foreign policy issues) on a scale from 1 (*extremely conservative*) to 7 (*extremely liberal*). Responses (Cronbach’s α = 0.95) were averaged to create a composite political ideology score. Participants indicated which of the two major political parties best “fit” them (*N*_*Republican*_ = 45; *N*_*Democrat*_ = 105).

#### Perceived partisan threat

Participants indicated perceived partisan threat over four items: “How much of a threat do you think a person of the following party [Republican, Democrat, Having no political affiliation] poses to you [society]?” and “How much of a threat do you think a person of the following party *who is also an elected official* poses to you [society]?” on scales from 1 *(not at all)* to 7 *(very much)*. Responses (Cronbach’s αs at least 0.92) were averaged to create three composite scores.

#### Perceiver partisan affiliation

Participants rated affiliation (“How strongly do you feel affiliated with these political categories?”) with Republicans, Democrats, and people with no political affiliation on scales from 1 *(not affiliated at all)* to 7 *(strongly affiliated)*.

Lastly, participants provided demographic information.

## Results

### Analytic strategy

We used the base R function *lm* for linear regressions and the lme4 package^43^ for mixed effects models. We used lmerTest^44^ to calculate model *p*-values. We used *tab_model* from the sjPlot package to create publication quality model result tables with standardized estimates and confidence intervals^45^. We used the *emtrends* and *eff_size* functions from the emmeans package^46^ to calculate simple slopes to characterize interaction effects. Power estimates were generated via the simr package^47^. We calculated confidence intervals via the base R function *confint*. For both experiments, analyzed datasets and code are available in the Open Science Framework repository (https://osf.io/xdf6m/?view_only=a97a8ee15b044bcfbc0cbe7715ff610a).

### Characterizing partisanship effects on the relation between facial trustworthiness and evaluated likability

We fit a linear mixed-effect model regressing likability on Partisan Label (Republican, Democrat, unlabeled), Facial Trustworthiness (standardized), Perceiver Political Ideology (standardized), and their interactions as fixed effects. See Table 2 for coefficient information. The model included a random effects structure such that intercepts varied by participant and by face identity. It also varied interactive effects of Partisan Label and Facial Trustworthiness by participant and Partisan Label effects by face identity. Varying interactive effects facilitated exploratory analyses (see below). Models with more parsimonious structures yielded similar results. The reference level for Partisan Label across experiments was Democrat. This choice was due to our expectation that the sample would skew ideologically liberal. Thus, we might expect coefficients reflecting partisanship effects on evaluations to appear strongest when contrasted with evaluations of Democrats. Note, however, that this reference level choice does not affect the nature of any emergent patterns.

**Table 2:** Mixed effects model predicting likability in experiment 1.

Predictors	Evaluated likability				
	Estimates	CI	β	Std. CI	p
Intercept	3.57	3.38 to 3.75	0.08	− 0.04 to 0.20	< 0.001
Party label [unlabeled]	− 0.10	− 0.19 to − 0.02	− 0.07	− 0.12 to − 0.01	0.015
Party label [Republican]	− 0.27	− 0.41 to − 0.12	− 0.18	− 0.27 to − 0.08	< 0.001
Political ideology	0.18	0.01 to 0.35	0.12	0.01 to 0.23	0.034
Facial trustworthiness	0.30	0.21 to 0.39	0.20	0.14 to 0.26	< 0.001
Party label [unlabeled] × political ideology	− 0.23	− 0.32 to − 0.15	− 0.15	− 0.21 to − 0.10	< 0.001
Party label [Republican] × political ideology	− 0.49	− 0.63 to − 0.34	− 0.32	− 0.42 to − 0.22	< 0.001
Party label [unlabeled] × facial trustworthiness	0.03	− 0.02 to 0.08	0.02	− 0.02 to 0.05	0.301
Party label [Republican] × facial trustworthiness	− 0.02	− 0.07 to 0.02	− 0.02	− 0.05 to 0.01	0.322
Political ideology × facial trustworthiness	0.01	− 0.03 to 0.05	0.01	− 0.02 to 0.03	0.609
(Party label [unlabeled] × political ideology) × facial trustworthiness	− 0.06	− 0.11 to − 0.01	− 0.04	− 0.07 to − 0.01	0.015
(Party label [Republican] × political ideology) × facial trustworthiness	− 0.07	− 0.11 to − 0.02	− 0.05	− 0.07 to − 0.02	0.002

#### Partisanship shifts positive facial trustworthiness effects on likability

**Figure 2:**
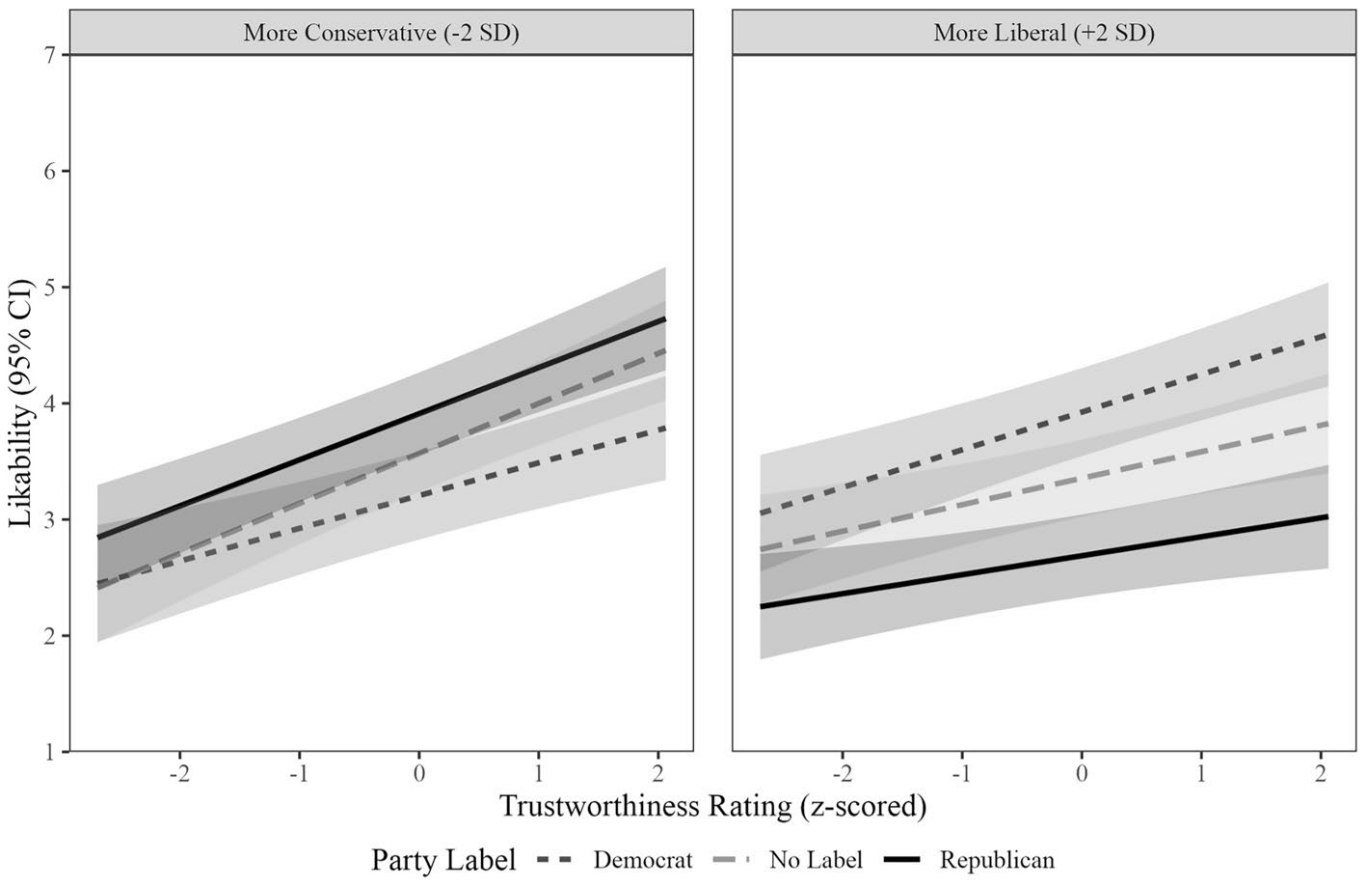
Similar relative to opposing partisan labels accentuated positive facial trustworthiness effects on likability in Experiment 1.

Three-way interactions between Partisan Label, Facial Trustworthiness, and Perceiver Political Ideology showed that partisanship *altered* facial trustworthiness effects on likability (Fig. 2; see Table 3 for simple slope information; see Table 4 for descriptive and inferential statistics). At the reported effect sizes and alpha = 0.05, we had 93% sensitivity to detect these interactions. We characterized interactions by examining differences in the strength of facial trustworthiness effects on evaluated likability at each Partisan Label among partisans. A one-sample t-test against the scale midpoint (4) showed the mean composite political ideology score skewed liberal, *t*(149) = 5.22, *p* < 0.001, *d* = 0.43. We thus examined effects two standard deviations below (more conservative) and above (more liberal) the mean to better reflect partisans’ evaluations.

**Table 3:** Slopes between facial trustworthiness and likability by party label and political ideology (+ /− 2SD) in experiment 1.

Experiment 1		
Party label	Political ideology	Estimated slope [95% CI]
Democrat	Conservative	0.28 [0.16, 0.40]
Republican	Conservative	0.40 [0.28, 0.51]
None	Conservative	0.43 [0.30, 0.56]
Democrat	Liberal	0.32 [0.20, 0.44]
Republican	Liberal	0.16 [0.05, 0.28]
None	Liberal	0.23 [0.10, 0.36]

**Table 4:** Comparisons of evaluated liability of partisan groups by political ideology (+ /– 2SD) and facial trustworthiness (+ /− 1SD) in experiment 1.

More conservative participants (− 2SD perceiver political ideology)						
	Republican label estimate (SE)	Unlabeled estimate (SE)	Democrat label estimate (SE)	Republican vs. democrat	Republican vs. unlabeled	Democrat vs. unlabeled
Less facial trustworthiness	3.41 (0.20)	3.14 (0.18)	2.92 (0.20)	*z* = 3.43, *p* = 0.001	*z* = 3.52, *p* < 0.001	*z* = 2.08, *p* = 0.04
More facial trustworthiness	4.31 (0.20)	4.00 (0.18)	3.49 (0.20)	*z* = 4.50, *p* < 0.001	*z* = 0.31, *p* = 0.01	*z* = 4.37, *p* < 0.001

More liberal participants (+ 2SD perceiver political ideology)						
	Republican label estimate (SE)	Unlabeled estimate (SE)	Democrat label estimate (SE)	Republican vs. democrat	Republican vs. unlabeled	Democrat vs. unlabeled
Less facial trustworthiness	2.53 (0.19)	3.13 (0.18)	3.60 (0.20)	*z* = 6.27, *p* < 0.001	*z* = 5.65, *p* < 0.001	*z* = 4.58, *p* < 0.001
More facial trustworthiness	2.85 (0.20)	3.58 (0.18)	4.25 (0.20)	*z* = 7.67, *p* < 0.001	*z* = 6.03, *p* < 0.001	*z* = 5.70, *p* < 0.001

For more conservative perceivers, the facial trustworthiness effect on likability was stronger for Republican- than Democrat-labeled faces, *b* = 0.11, *z* = 2.25, *p* = 0.02. It was also stronger for unlabeled than Democrat-labeled faces, *b* = 0.14, *z* = 2.65, *p* = 0.008. No difference in strength emerged between Republican-labeled and unlabeled faces, *b* = 0.03, *z* = 0.62, *p* = 0.54.

For more liberal perceivers, the effect was stronger for Democrat- than Republican-labeled faces, *b* = 0.16, *z* = 3.16, *p* = 0.002. Though in the expected direction, it was not stronger for Democrat-labeled than unlabeled faces, *b* = 0.10, *z* = 1.72, *p* = 0.08. No difference in strength emerged between Republican-labeled and unlabeled faces, *b* = − 0.06, *z* = 1.19, *p* = 0.23.

#### Partisanship affects likability overall

Two-way interactions between Perceiver Political Ideology and Partisan Label confirmed that partisanship affected likability. We characterized interactions by examining Political Ideology effects at each Partisan Label. Having a more liberal ideology related to evaluating Republican-labeled faces as less, *b* = − 0.31, *SE* = 0.08, *z* = 3.84, *p* < 0.001, and Democrat-labeled faces as more, *b* = 0.18, *SE* = 0.08, *z* = 2.12, *p* = 0.03, likable. No effect emerged for unlabeled faces, *b* = − 0.05, *SE* = 0.07, *z* = 0.72, *p* = 0.47. All slopes differed in strength, *z*s > 5.48, *p*s < 0.001.

### Exploratory analyses assessing a potential role of perceived partisan threat

#### Perceiver ideology effects on perceived partisan threat

We confirmed that perceiver political ideology mapped onto perceived partisan threat by regressing composite political ideology score (standardized) on the composite partisan threat ratings (Republican, Democrat, and none). The model was significant, *F*(5, 444) = 51.26, *p* < 0.001, R^2^ = 0.36. Having a more liberal ideology related to perceiving Democrats as less threatening, *b* = − 0.92, *t* = 7.73, *p* < 0.001, Republicans as more threatening, *b* = 1.23, *t* = 10.29, *p* < 0.001, and did not significantly relate perceiving threat from people with no party affiliation, *b* = − 0.12, *t* = 1.01, *p* = 0.31.

#### Exploring partisan threat as affecting the relation between facial trustworthiness and likability

We next tested whether perceived partisan threat related to the difference in facial trustworthiness effects between partisan faces. Specifically, we correlated the random slopes for the interaction between facial trustworthiness effects for Republican- versus Democrat-labeled faces with composite threat scores from Democrats and Republicans. Random slopes reflect how much each person deviated from the population-level estimate. We added the fixed effect for this interaction (i.e. the population-level estimate) to each random slope so values could be interpreted in the context of the fixed effect. A positive correlation indicates that perceived threat of a partisan group (Democrats or Republicans) relates to a stronger effect of facial trustworthiness on likability for Republican- than Democrat-labeled faces.

**Figure 3:**
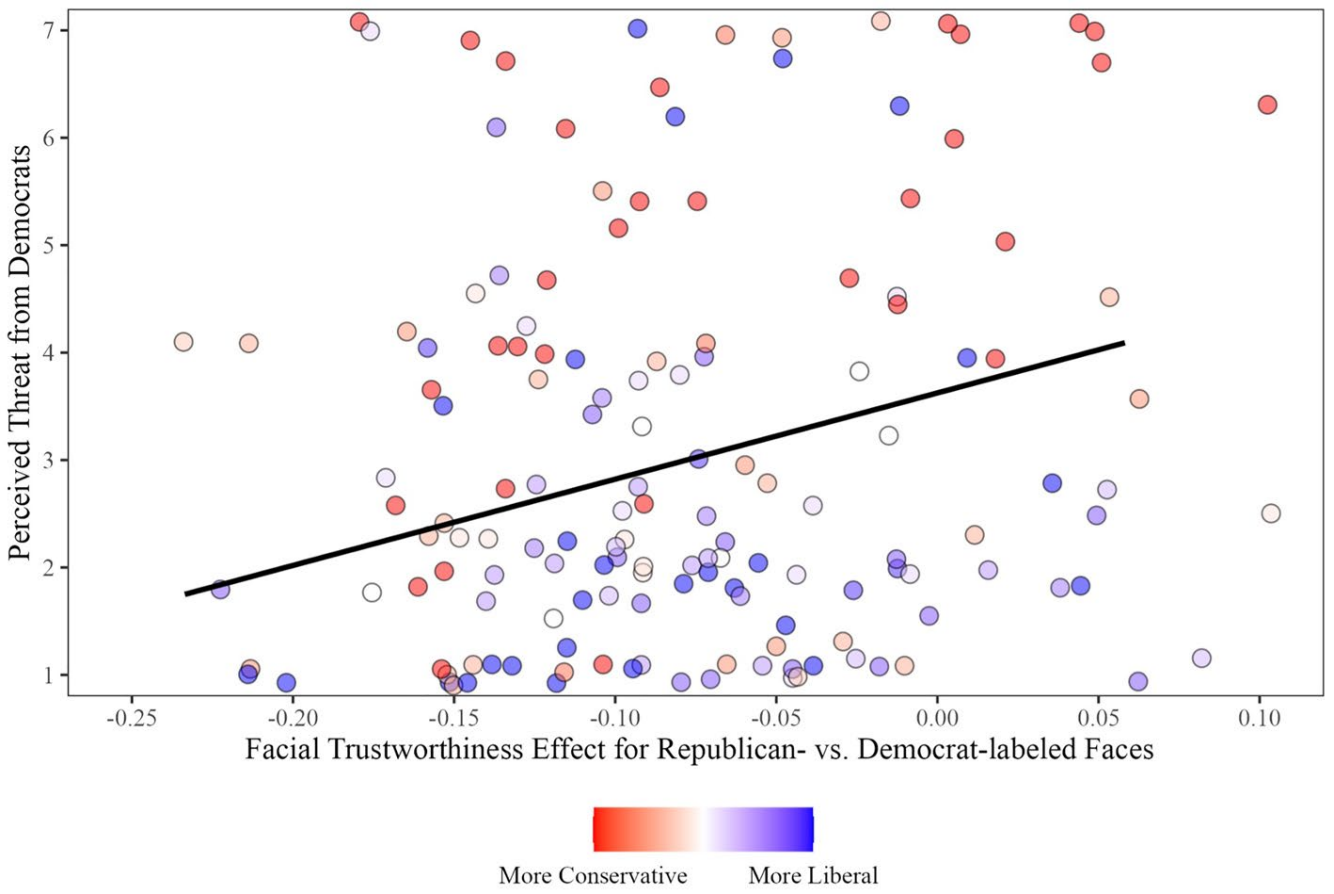
A stronger positive facial trustworthiness effect for Republican-labeled than Democrat-labeled faces positively related to perceived threat from Democrats in Experiment 1. Participants’ own political ideology (standardized composite scores) is represented by the color of the dots.

A positive relation between perceived threat from Democrats and the interactive effect emerged, *r*(148) = 0.18, *p* = 0.02, 95% CI [0.02, 0.33] (Fig. 3). People more threatened by Democrats had a stronger Facial Trustworthiness effect on likability for Republican- than Democrat-labeled faces. Although in the expected negative direction, no significant relation emerged between perceived threat from Republicans and the interactive effect, *r*(148) = − 0.11, *p* = 0.16, 95% CI [− 0.27, 0.05].

## Discussion

Extending work showing that partisan labels polarize impressions of partisan faces^10–12^, partisan labels *shifted* the positive relation between facial trustworthiness and evaluated likability. Consistent with the salience of higher facial trustworthiness to ingroups^31^, facial trustworthiness more strongly related to the likability of shared relative to opposing partisan faces across more conservative and liberal perceivers. Extending work showing political beliefs to elicit affectively polarized impressions^5,8^, partisanship *alters* contributions of facial trustworthiness to likability impressions. Growing affective polarization^39^ and sectarianism^1^ may thus be reflected in how facial trustworthiness links to impressions. Because shared relative to opposing partisans were always evaluated as being more likable even when faces were relatively lower in facial trustworthiness, these findings suggest that higher facial trustworthiness accentuates a tendency for people to make broadly more positive evaluations of shared relative to opposing partisans.

Patterns of relation strength relative to unlabeled faces revealed nuanced effects. For more conservative perceivers, the relation was stronger for unlabeled than Democrat-labeled faces. It was not stronger for Republican-labeled than unlabeled faces. A stronger pattern for Republican- *and* unlabeled faces than Democrat-labeled faces suggests that higher facial trustworthiness among opposing partisans was not valued as much as it was in other faces. These findings may reflect a perspective that conservatives are particularly sensitive to threat^48^ that extends to face perception. When conservatives are given a threatening partisan label, a positive cue on a paired face may not heavily weigh into evaluations. Indeed, the relation was not stronger for Republican-labeled than unlabeled faces, which were unassociated with perceived partisan threat. Indeed, people behave more favorably toward independent than opposing partisans^5^. Moreover, people more threatened by Democrats had a stronger positive relation between facial trustworthiness and the evaluated likability of Republican- than Democrat-labeled faces. Partisan threat may shape the extent to which higher facial trustworthiness accentuates the evaluated likability of shared relative to opposing partisans, at least among conservatives.

Unlike more conservative perceivers, more liberal perceivers had no difference in the relation between opposing partisan and unlabeled faces. More liberal perceivers thus did not exhibit broad partisan derogation. Like more conservative perceivers, more liberal perceivers exhibited similar relation strength for shared and unlabeled faces, though effects were in the direction of being stronger for shared partisans. Speculatively, that these effects were in the direction of being consistently stronger for shared partisans suggests that more liberal perceivers may be more motivated by ingroup favoritism than are conservative perceivers. Indeed, ingroup favoritism often motivates behavior more strongly than outgroup derogation^49,50^. Conservatives’ attitudes are theorized to serve an ego-defensive function against threat^48^. Speculatively, liberals might react to faces in ways that enhance similar partisans at the expense of others rather than a reaction to broadly derogate opposing partisans^51^. Such a finding does not rule out prejudicial behavior from liberals. Rather, it suggests that threat may not be a focal mechanism in the current data. Ingroup favoritism, for instance, has been proposed to be a more significant basis for discrimination in America than outgroup hostility^52^.

Experiment 1 showed that shared relative to opposing partisanship strengthens the relation between facial trustworthiness and likability. Experiment 2 was designed to replicate and extend this finding. First, we sought to replicate this finding using a larger sample to show its reliability. Second, we used an experimental manipulation to explore whether the above-described patterns may depend on an inter-party threat emphasis.

## Experiment 2

People often see life as zero-sum, whereby gains of one group are matched by losses of another^53^. This thinking perpetuates intergroup conflict^54^ and is pervasive in politics^55^. Contexts where resources are scarce (relative to abundant) emphasize this thinking, engendering mistrust and discriminatory behavior^56^. To examine whether the key finding from Experiment 1 had a basis in perceived threat, we emphasized inter-party threat by having people expect (versus not expect) some partisans to lose resources to other partisans.

We manipulated an inter-party threat emphasis by varying the resources liberals would have expected after gains by the Republican party. We chose this manipulation given the proximity of the experiment to the 2022 midterm elections in the United States and given a history of the incumbent president’s party losing ground during midterms. Perceived Republican threat did not significantly relate to the interactive effect in Experiment 1. In Experiment 2, we focused the manipulation on *liberals’* scarcity and abundance to understand contexts in which liberals may also be motivated by threat. In the liberal scarcity condition, people learned more federal assets would go to Republican-advocated causes and there would not be enough for Democrat-advocated causes. In the liberal abundance condition, people learned that although more federal assets would go to Republican-advocated causes, there would be resources for Democrat-advocated causes given local governments comprised of Democrats.

Because affective polarization characterizes partisan relations in the United States^8^, we reasoned that people may naturally think about partisans in terms of conflict and inter-party threat^39^. If true, partisanship effects on the relation between facial trustworthiness and likability in Experiment 1 may reflect effects dependent on an inter-party conflict and threat emphasis. We thus expected that emergent patterns in the liberal scarcity condition would mirror the patterns described in Experiment 1. Given this expectation, we anticipated that these patterns should weaken in a context emphasizing an abundance of partisan resources.

## Method

### Participants

Experiment 2 comprised a design assessing interactive effects of resource scarcity (scarcity, abundance), partisan label (Republican, Democrat, unlabeled), facial trustworthiness (continuously measured), and perceiver political ideology (continuously measured). We targeted the sample size for the experiment based on an expected four-way interaction between these variables but did not have a point of reference for the size of this effect. To this end, we doubled the sample from Experiment 1 to ensure at least 125 people each in the scarcity and abundance conditions, oversampling for the same reasons. Overall, 300 people from the United States were recruited from MTurk. Two were excluded for not providing relevant vignette responses (see below), yielding an analyzed sample of 298 people (*M*_*age*_ = 38.13, *SD* = 11.39; *M*_*years of education*_ = 15.46, *SD* = 2.50; 139 identifying as female). Of these people, 230 identified as White, 28 as Asian, 27 as Black, 10 as multi-racial, 2 as American Indian/Alaska native, and 1 self-reported having an unknown race. Of these people, 276 identified as non-Hispanic.

### Task

The task replicated Experiment 1 with the following changes. People were randomly assigned to scarcity (*N* = 136) or abundance (*N* = 162) conditions. People in the scarcity (*M* = 4.72, *SD* = 1.65) and abundance (*M* = 4.65, *SD* = 1.68) conditions had similar composite political ideology, *t*(296) = 0.36, *p* = 0.72, *d* = 0.04. People were told the experiment consisted of two tasks. They read, “In the first task, you will read an article about the growing number of Republican voters in America. Read this article carefully. After you read this article, you will be asked to provide one to two sentences in your own words about the conclusion of the article. You will be able to press the arrow to advance from the article screen after 30 s have passed. Later, we will ask you questions about your knowledge of the article”.

The article was adapted from a prior manipulation^57^. The first paragraph read, “WASHINGTON, DC (AP)—The proportion of Republicans who are expected to vote in the 2022 midterm election is increasing, the U.S. Census Bureau said Wednesday. There are now 49% of people that define themselves to be Republicans and voted in the past election in the U.S., the bureau reported. Reportedly, this number is expected to increase within the next 10 years. By 2030, Republicans are predicted to form a full 56% of expected voters. Projections also predict 59% of Republican Americans will be active voters in the U.S. elections by 2050. Thus, most active American voters in the coming decades are expected to be Republicans”.

The second paragraph for the scarcity condition read, “Though some experts are optimistic that a more politically homogeneous government will work more smoothly, far more believe that there will not be enough governmental resources to accommodate the goals of both the Republican and Democrat parties. ‘Unfortunately, Democrats should suffer the most from these demographic trends,’ said Dr. Kenneth Fields, a research professor at Georgetown University’s Center for Population and Government. ‘With more federal assets going to Republican-advocated causes, there simply won’t be as much to go around for other causes’.”.

The second paragraph for the abundance condition read, “Though some experts are pessimistic that a more politically homogeneous government will work more smoothly, far more believe that there will be enough governmental resources to accommodate the goals of both the Republican and Democrat Parties. ‘Fortunately, Democrats shouldn’t suffer much from these demographic trends,’ said Dr. Kenneth Fields, a research professor at Georgetown University’s Center for Population and Government. ‘Local governments comprised of Democrats will still allocate assets toward Democrat-advocated causes, suggesting there will be enough resources to support them’.”.

People wrote one to two sentences about the conclusion. Two were excluded for irrelevant responses. People in the scarcity (*M*_*seconds*_ = 138.47, *SD* = 115.01) and abundance (*M*_*seconds*_ = 150.30, *SD* = 107.11) conditions spent a similar amount of time on the article screen, *t*(296) = 0.92, *p* = 0.36, *d* = 0.11.

They next read, “Welcome to the second task. We are creating a face database of student faces to use in future lab research. When creating this database, we need to collect information on how people evaluate the faces.” They then completed the Experiment 1 task and indicated adherence (*M* = 1.20, *SD* = 0.70). All indicated they rated faces. Finally, they saw the article again and rated how threatened they, Republicans, and Democrats would be by its conclusion on scales from 1 (*not at all threatened*) to 7 (*extremely threatened*).

### Partisanship characterization

Responses to political ideology items (Cronbach’s α = 0.94) were averaged. People indicated which major party best “fit” them (*N*_*Republican*_ = 94; *N*_*Democrat*_ = 203). One did not respond. Responses to threat items (Cronbach’s αs at least 0.91) were averaged to make composite scores. See Table 5 for descriptives and intercorrelations.

**Table 5:** Intercorrelations between partisanship characterization measures in experiment 2.

Variable	Scarcity M (SD)	Abundance M (SD)	1	2	3	4	5	6	7
1. Political ideology	4.72 (1.65)	4.65 (1.68)		0.59** [0.48, 0.68]	− 0.01 [− 0.16, 0.15]	− 0.50** [− 0.61, − 0.37]	− 0.73** [− 0.80, − 0.65]	− 0.01 [− 0.16, 0.15]	0.67** [0.57, 0.74]
2. Perceived threat (Republican)	3.94 (1.83)	3.94 (1.85)	0.68** [0.58, 0.76]		0.28** [0.13, 0.42]	− 0.09 [− 0.25, 0.06]	− 0.52** [− 0.62, − 0.39]	0.08 [− 0.08, 0.23]	0.56** [0.44, 0.66]
3. Perceived threat (no party)	2.63 (1.34)	2.51 (1.23)	− 0.04 [− 0.20, 0.13]	0.16 [− 0.01, 0.32]		0.40** [0.27, 0.53]	0.20* [0.04, 0.34]	0.03 [− 0.13, 0.18]	0.22** [0.07, 0.36]
4. Perceived threat (democrat)	3.34 (1.74)	3.21 (1.71)	− 0.39** [− 0.52, − 0.24]	− 0.20* [− 0.36, − 0.04]	0.51** [0.37, 0.62]		0.54** [0.41, 0.64]	0.22** [0.07, 0.37]	− 0.45** [− 0.57, − 0.32]
5. Affiliation (Republican)	2.46 (1.98)	2.48 (1.97)	− 0.67** [− 0.75, − 0.56]	− 0.53** [− 0.64, − 0.40]	0.29** [0.12, 0.43]	0.46** [0.32, 0.59]		− 0.16* [− 0.31, − 0.01]	− 0.46** [− 0.57, − 0.33]
6. Affiliation (no party)	3.55 (2.14)	3.65 (2.11)	0.01 [− 0.16, 0.18]	0.03 [− 0.14, 0.20]	− 0.02 [− 0.19, 0.14]	0.20* [0.03, 0.35]	− 0.12 [− 0.28, 0.05]		− 0.26** [− 0.40, − 0.11]
7. Affiliation (democrat)	3.67 (2.12)	3.67 (2.18)	0.63** [0.52, 0.72]	0.53** [0.40, 0.64]	0.13 [− 0.04, 0.29]	− 0.44** [− 0.57, − 0.29]	− 0.41** [− 0.54, − 0.26]	− 0.23** [− 0.39, − 0.07]	

*M* and *SD* are used to represent mean and standard deviation, respectively.

Values in square brackets indicate the 95% confidence interval for each correlation. Participants in scarcity and abundance conditions are represented, respectively, below and above the diagonal.

* indicates *p* < 0.05.

** indicates *p* < 0.01.

## Results

### Characterizing partisanship and resource scarcity effects on the relation between facial trustworthiness and likability

We fit a linear mixed-effect model regressing likability on Partisan Label (Republican, Democrat, unlabeled), Facial Trustworthiness (standardized), Perceiver Political Ideology (standardized), Resource Scarcity (scarcity and abundance), and their interactions as fixed effects. See Table 6 for coefficient information. The random effects structure specified intercepts varying by participant and face identity. Interactive Partisan Label and Facial Trustworthiness effects varied by participant and Partisan Label effects varied by face identity.

**Table 6:** Mixed effects model predicting likability in experiment 2.

Predictors	Estimates	CI	Evaluated likability		
			β	Std. CI	p
(Intercept)	3.49	3.32 to 3.67	− 0.05	− 0.17 to 0.06	< 0.001
Resource scarcity [abundance]	0.22	0.00 to 0.44	0.15	0.00 to 0.30	0.049
Party label [unlabeled]	0.02	− 0.06 to 0.10	0.01	− 0.04 to 0.07	0.665
Party label [Republican]	− 0.05	− 0.21 to 0.11	− 0.03	− 0.14 to 0.07	0.524
Political ideology	0.25	0.09 to 0.41	0.17	0.06 to 0.28	0.003
Facial trustworthiness	0.26	0.18 to 0.34	0.17	0.12 to 0.23	< 0.001
Resource scarcity [abundance] × party label [unlabeled]	− 0.05	− 0.16 to 0.06	− 0.03	− 0.10 to 0.04	0.394
Resource scarcity [abundance] × party label [Republican]	− 0.14	− 0.36 to 0.07	− 0.09	− 0.24 to 0.05	0.201
Resource scarcity [abundance] × political ideology	− 0.19	− 0.41 to 0.03	− 0.13	− 0.28 to 0.02	0.084
Party label [unlabeled] × political ideology	− 0.17	− 0.25 to − 0.09	− 0.12	− 0.17 to -0.06	< 0.001
Party label [Republican] × political ideology	− 0.41	− 0.57 to − 0.25	− 0.27	− 0.38 to − 0.17	< 0.001
Resource scarcity [abundance] × facial trustworthiness	0.07	0.01 to 0.13	0.04	0.00 to 0.08	0.033
Party label [unlabeled] × facial trustworthiness	0.04	− 0.01 to 0.09	0.03	− 0.01 to 0.06	0.116
Party label [Republican] × facial trustworthiness	0.00	− 0.05 to 0.05	0.00	− 0.03 to 0.03	0.907
Political ideology × facial trustworthiness	0.04	− 0.00 to 0.09	0.03	− 0.00 to 0.06	0.054
(Resource scarcity [abundance] × Party label [unlabeled]) × political ideology	0.01	− 0.10 to 0.12	0.01	− 0.07 to 0.08	0.879
(Resource scarcity [abundance] × party label [Republican]) × political ideology	− 0.02	− 0.23 to 0.20	− 0.01	− 0.16 to 0.13	0.869
(Resource scarcity [Abundance] × Party label [unlabeled]) × Facial trustworthiness	− 0.07	− 0.14 to − 0.00	− 0.05	− 0.09 to − 0.00	0.048
(Resource scarcity [abundance] × party label [Republican]) × facial trustworthiness	− 0.07	− 0.13 to − 0.00	− 0.04	− 0.09 to 0.00	0.049
(Resource scarcity [abundance] × political ideology) × Facial trustworthiness	− 0.01	− 0.07 to 0.05	− 0.01	− 0.05 to 0.03	0.736
(Party label [unlabeled] × Political ideology) × Facial trustworthiness	− 0.08	− 0.14 to − 0.03	− 0.06	− 0.09 to − 0.02	0.002
(Party label [Republican] × Political ideology) × facial trustworthiness	− 0.05	− 0.10 to − 0.01	− 0.04	− 0.07 to − 0.00	0.028
(Resource scarcity [abundance] × Party label [unlabeled] × political ideology) × facial trustworthiness	0.07	0.00 to 0.14	0.05	0.00 to 0.09	0.047
(Resource scarcity [abundance] × party label [Republican] × political ideology) × facial trustworthiness	0.04	− 0.03 to 0.10	0.02	− 0.02 to 0.07	0.282

#### Replicating and extending experiment 1: resource scarcity qualifies partisanship effects on the relation between facial trustworthiness and likability

**Figure 4:**
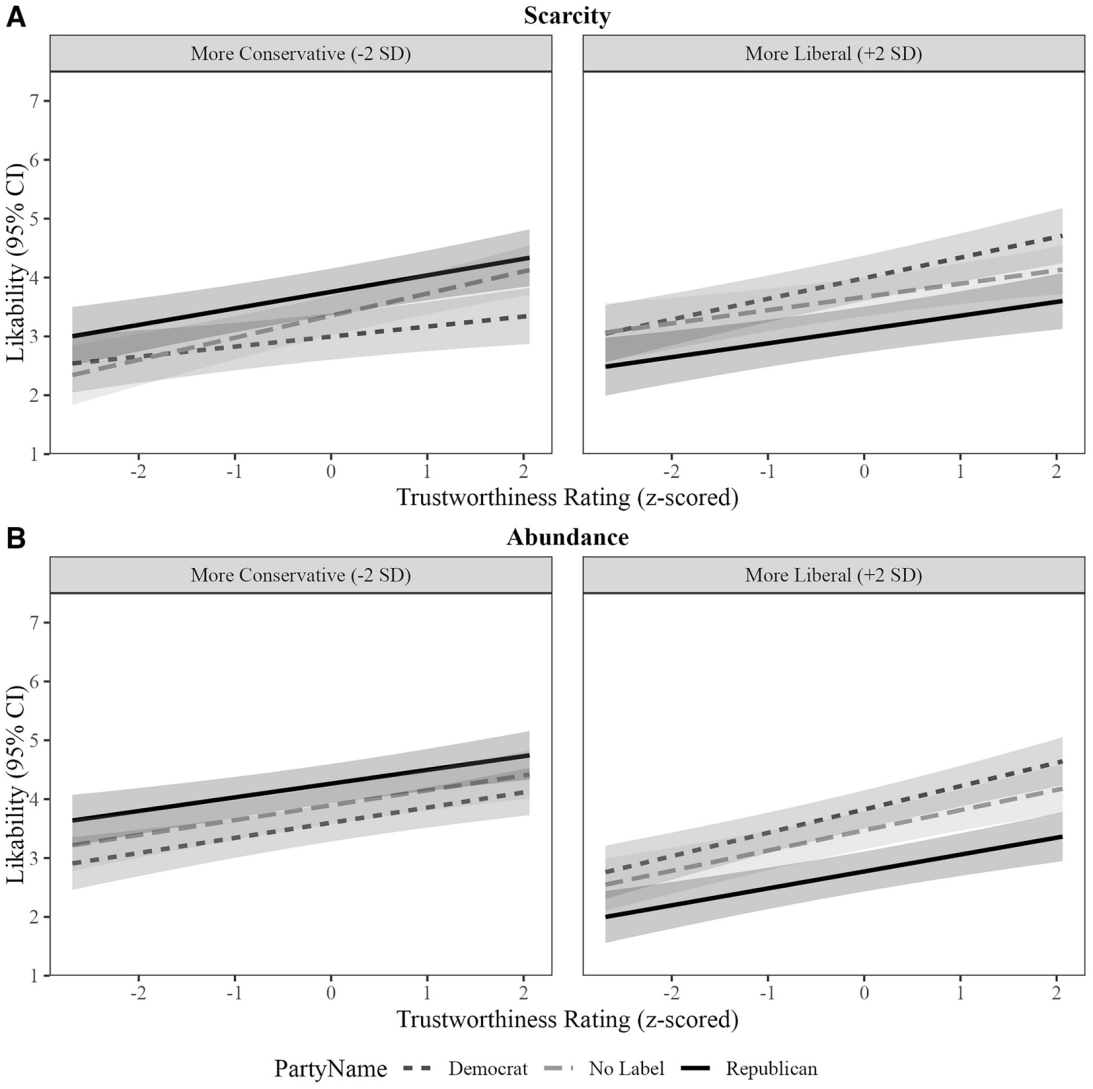
In Experiment 2, perceiver and target partisanship largely interacted to effect facial trustworthiness effects on likability in the scarcity (**A**) but not the abundance (**B**) condition.

We expected resource scarcity to qualify the three-way interactions from Experiment 1. Resource scarcity qualified the interaction for unlabeled relative to Democrat-labeled faces. For completeness, we describe patterns for unlabeled relative to Democrat-labeled faces and for Republican- relative to Democrat-labeled faces (Fig. 4; see Table 7 for simple slopes details). For interpretability, we describe patterns as in Experiment 1 for each condition. At the reported effect sizes and alpha = 0.05, we had 100% sensitivity to detect these focal interactions.

**Table 7:** Slopes between facial trustworthiness and likability by scarcity manipulation, party label, political ideology (+ /– 2 SD) in experiment 2.

Experiment 2			
Manipulation	Party label	Political ideology	Estimated slope [95% CI]
Scarcity	Democrat	Conservative	0.17 [0.04, 0.29]
Scarcity	Republican	Conservative	0.28 [0.16, 0.40]
Scarcity	None	Conservative	0.38 [0.25, 0.51]
Abundance	Democrat	Conservative	0.26 [0.14, 0.37]
Abundance	Republican	Conservative	0.23 [0.12, 0.34]
Abundance	None	Conservative	0.25 [0.13, 0.37]
Scarcity	Democrat	Liberal	0.35 [0.23, 0.47]
Scarcity	Republican	Liberal	0.24 [0.12, 0.36]
Scarcity	None	Liberal	0.22 [0.09, 0.35]
Abundance	Democrat	Liberal	0.39 [0.28, 0.51]
Abundance	Republican	Liberal	0.29 [0.18, 0.41]
Abundance	None	Liberal	0.34 [0.22, 0.46]

##### Scarcity condition

As expected, patterns from the scarcity condition largely paralleled Experiment 1. For more conservative perceivers, a stronger facial trustworthiness effect emerged for Republican- than Democrat-labeled faces, *b* = − 0.11, *z* = 1.99, *p* = 0.046, and for unlabeled than Democrat-labeled faces, *b* = − 0.21, *z* = 3.50, *p* = 0.001. No effect strength difference emerged between Republican-labeled and unlabeled faces, *b* = 0.10, *z* = 1.69, *p* = 0.09.

For more liberal perceivers, the facial trustworthiness effect was marginally stronger for Democrat- than Republican-labeled faces, *b* = 0.11, *z* = 1.93, *p* = 0.05, and for Democrat-labeled than unlabeled faces, *b* = 0.13, *z* = 2.15, *p* = 0.03. No effect strength difference emerged between Republican-labeled and unlabeled faces, *b* = − 0.02, *z* = 0.34, *p* = 0.73.

##### Abundance condition

Patterns from the abundance condition contrasted Experiment 1. For more conservative perceivers, the facial trustworthiness effect was not stronger for Republican- than Democrat-labeled faces, *b* = − 0.02, *z* = 0.35, *p* = 0.73, and there was no difference in effects for Democrat-labeled and unlabeled faces, *b* = 0.003, *z* = 0.06, *p* = 0.96. Like Experiment 1 and the scarcity condition, no effect strength difference emerged between Republican-labeled and unlabeled faces, *b* = 0.02, *z* = 0.46, *p* = 0.65.

For more liberal perceivers, facial trustworthiness effects were marginally stronger for Democrat- than Republican-labeled faces, *b* = 0.10, *z* = 1.97, *p* = 0.05. Unlike Experiment 1 and the scarcity condition, they were not stronger for Democrat-labeled than unlabeled faces, *b* = 0.05, *z* = 1.01, *p* = 0.31. Like Experiment 1 and the scarcity condition, no effect strength difference emerged between Republican-labeled and unlabeled faces, *b* = 0.05, *z* = 0.90, *p* = 0.37.

#### Replicating experiment 1: partisanship affects likability overall

Two-way interactions again emerged. Having a more liberal ideology related to evaluating Republican-labeled faces as less, *b* = − 0.26, *SE* = 0.06, *z* = 4.59, *p* < 0.001, and Democrat-labeled faces as more, *b* = 0.15, *SE* = 0.06, *z* = 2.72, *p* = 0.007, likable. A non-significant relation emerged for unlabeled faces, *b* = − 0.01, *SE* = 0.05, *z* = 0.31, *p* = 0.76. These slopes all differed in strength, *z*s > 6.07, *p*s < 0.001.

### Validating that the resource scarcity manipulation related to threat

#### Threat to democrats

One sample t-tests against the scale midpoint (4) showed the scarcity (*M* = 5.83, *SD* = 1.47), *t*(135) = 14.49, *p* < 0.001, *d* = 1.24, and abundance (*M* = 4.97, *SD* = 1.78), *t*(161) = 6.91, *p* < 0.001, *d* = 0.54, articles were more than moderately threatening to Democrats.

A model regressing article-related threat to Democrats on Political Ideology (standardized), Resource Scarcity, and the interaction, was significant, *F*(3, 294) = 10.42, *p* < 0.001, R^2^ = 0.09. A Political Ideology effect, *b* = 0.22, *t* = 2.32, *p* = 0.02, showed that having a more liberal ideology related to evaluating articles as more threatening to Democrats. Validating the manipulation, a Resource Scarcity effect, *b* = − 0.43, *t* = 4.51, *p* < 0.001, showed that people in the scarcity relative to the abundance condition evaluated the article as more threatening to Democrats. Although these contexts were both threatening, the extent of threat differed.

An interaction emerged, *b* = − 0.19, *t* = 2.00, *p* = 0.047. For the abundance, *b* = 0.41, *t* = 3.23, *p* = 0.001, but not scarcity, *b* = 0.03, *t* = 0.22, *p* = 0.83, condition, having a more liberal ideology related to evaluating the article as more threatening to Democrats. No effect in the scarcity condition reflects higher overall threat for Democrats in that condition. A positive effect in the abundance condition reflects that partisans perceive threat even when contexts are not zero-sum.

#### Threat to republicans

One sample t-tests against the midpoint showed the scarcity (*M* = 1.65, *SD* = 1.33), *t*(135) = 20.62, *p* < 0.001, *d* = 1.78, and abundance (*M* = 1.90, *SD* = 1.36), *t*(161) = 19.62, *p* < 0.001, *d* = 1.54, articles were less than moderately threatening to Republicans. Tests against the endpoint showed the scarcity, *t*(135) = 5.67, *p* < 0.001, *d* = 0.49, and abundance, *t*(161) = 8.43, *p* < 0.001, *d* = 0.66, articles presented some threat to Republicans. Threat in partisan contexts is thus not eliminated even to the party gaining power. Repeating the regression on article-related threat to Republicans yielded a non-significant model, *F*(3, 294) = 1.73, *p* = 0.16, R^2^ = 0.01. Article-related threat to Republicans did not differ by the scarcity manipulation.

#### Threat to self

Repeating the regression on article-related threat to the self yielded a significant model, *F*(3, 294) = 55.03, *p* < 0.001, R^2^ = 0.35. A Political Ideology effect, *b* = 1.23, *t* = 12.74, *p* < 0.001, showed that having a more liberal ideology related to articles being more threatening to the self. Resource Scarcity, *b* = − 0.13, *t* = 1.36, *p* = 0.17, and interaction, *b* = − 0.17, *t* = 1.73, *p* = 0.08, effects were non-significant.

### Perceiver ideology effects on perceived partisan threat

Like Experiment 1, the model was significant, *F*(5, 886) = 84.31, *p* < 0.001, R^2^ = 0.31. Having a more liberal ideology related to perceiving Democrats as less threatening, *b* = − 0.77, *t* = 9.32, *p* < 0.001; Republicans as more threatening, *b* = 1.16, *t* = 13.99, *p* < 0.001; and did not significantly relate to perceiving threat from people with no party affiliation, *b* = − 0.03 *t* = − 0.33, *p* = 0.74. Including Resource Scarcity and its interactions did not explain more variance, *p* = 0.70.

## Discussion

Emphasizing more relative to less inter-party threat elicited differences in the relation between facial trustworthiness and evaluated likability. When observed alongside effects in the scarcity condition, the abundance condition seemed to reduce partisan differences in the facial trustworthiness-likability relation among more conservative participants. This pattern suggests that conservatives devalue higher trustworthiness on opposing partisan faces in a way that is consistent with well-documented conservative responses to threat^48^. That more conservative perceivers in the liberal scarcity condition had the same patterns between facial trustworthiness and evaluated likability as in Experiment 1 also suggests that even contexts where shared partisans make gains elicit partisanship effects on face impressions. Indeed, people are more likely to behave in affectively polarized ways in times of inter-party competition^58^ and are more willing to derogate opposing partisans when anticipating power shifts^59^. Although some work suggests that gaining political power decreases opposing partisan derogation in some circumstances^60^, our findings suggest that face impressions may be more resistant to this nuance.

More liberal perceivers showed a stronger facial trustworthiness-likability relation for shared versus opposing partisans regardless of condition. This finding may be attributed to the gains in Republican voter shares across conditions despite implications for liberal scarcity or abundance. Liberals in the scarcity condition had a weaker facial-trustworthiness-likability effect for unlabeled versus Democrat faces, whereas liberals in the abundance condition showed similar effects for these groups. These patterns raise the possibility more liberal perceivers broadly enhance high trustworthiness on shared partisan faces at the expense of others, potentially reflecting ingroup favoritism emergent at the expense of outgroups^52^. Overall, these patterns suggest distinct ways that people use facial trustworthiness in impressions of partisans.

Notably, more conservative and liberal perceivers in the abundance condition, but not the scarcity condition, had no difference between the relation for Democrat-labeled and unlabeled faces. These patterns suggest that strict partisan derogation and enhancement patterns weaken when de-emphasizing inter-party threat. It could be that evidence of partisanship is necessary to elicit differences in the facial trustworthiness-evaluated likability link. Speculatively, partisans may have less reason to treat unlabeled faces differently from shared and opposing partisan faces if a context does not elicit threat. Indeed, people are more likely to treat faces as outgroup members when contexts signal threat^61^. Higher trustworthiness on unlabeled faces may be treated differently from shared and opposing partisans based on context-dependent threat.

## General discussion

Extending work showing that partisanship affects the broad evaluation of facial characteristics^10–12^, a stronger positive relation emerged for shared relative to opposing partisan faces across more conservative and liberal perceivers across experiments. Exploratory analyses showed that partisanship effects may relate to the inter-party threat concomitant with affective polarization^39^. Specifically, partisan threat related to a stronger intergroup difference in this relation, at least among conservatives (Experiment 1). Patterns of opposing partisan derogation and shared partisan enhancement emergent across experiments also weakened when de-emphasizing inter-party threat (Experiment 2). Building on work showing higher trustworthiness to be salient for ingroup inclusion^31^, higher trustworthiness seems especially valued among shared partisans, a group by which people self-select and thus comprises faces widely ranging in trustworthiness. That faces appeared as shared and opposing partisans across task versions means that the faces themselves could not cause these patterns. Rather, these patterns parallel work showing that external information paired with faces affects their evaluation^35,36^.

The current findings suggest that partisanship affects how a core dimension of face perception^13^ contributes to impressions. Although a positive relation between facial trustworthiness and evaluated likability emerged for opposing partisans, a stronger relation consistently emerged for shared partisans. Because people are more likely to approach more positively perceived faces^18^, these findings raise the possibility that differences in this relation may add to inter-partisan tension by contributing to fewer opportunities for inter-partisan contact, at least when faces are relatively higher in facial trustworthiness. This possibility is important to examine given that this contact may be effective in reducing affective polarization^38^.

Exploratory analyses suggested that an emphasis on inter-party threat may exacerbate the salience of higher facial trustworthiness among shared partisans. These findings align with work showing that partisan threat polarizes partisan behavior^39^, extending it to person perception judgments. Whereas partisan threat affects face impressions of partisans^12^, the current work suggests that threat affects the *function* of partisanship in polarizing impressions. Speculatively, these findings suggest that de-emphasizing inter-party threat may be critical to elicit face impressions of partisans that facilitate interactions. Leveraging basic aspects of person perception may be important to reduce partisan animosity, where research has largely focused on higher-order interventions^62^. Future work may examine what cues need to be placed by opposing partisans to minimize opposing partisanship limiting the value of higher facial trustworthiness in impressions. Indeed, past work has leveraged learning paradigms to weaken stereotype effects on face impressions^63^.

One limitation of this work regards that it examined facial trustworthiness norms^42^ in relation to likability measured in actual participants. One possibility is that partisanship moderated the relation between facial trustworthiness and likability because people ignore facial characteristics when given opposing partisan cues. If true, it suggests little variability in trustworthiness judgments of opposing partisan faces. Future work may obtain both evaluations from participants and compare them to norms to determine whether people ignore facial characteristics of some partisans over others. Relatedly, future work might determine whether the relation between people’s evaluations of facial trustworthiness and obtained likability norms produce the same pattern of results. These analyses can determine whether a facial trustworthiness effect is stronger than a likability effect. It may be of theoretical interest given that people use the emotional structural resemblance underlying facial trustworthiness to infer others’ intentions^13,18^.

A second limitation regards the relatively small range of facial trustworthiness values among stimuli. This range was beneficial because people react more positively to more average versus more unusual faces^64^. However, this benefit means that whether the described findings generalize to faces more starkly contrasted on trustworthiness is unclear. Examining this possibility will be important to determine the efficacy of exaggerating one’s facial trustworthiness as a strategy to garner partisan support. Notably, we chose to quantify perceiver partisanship using a continuous measure of ideology. This choice allowed us to examine partisanship effects among more conservative and more liberal participants even though most participants identified as Democrat across experiments. Future work may obtain similarly large samples of self-identified Republicans and Democrats to more thoroughly explore how ingroup identification exaggerates a shift in the relation between facial trustworthiness and likability. For example, it could be that such exaggerations are strongest among Republicans who identify as more extremely ideologically conservative.

An open question regards whether the described patterns reflect a specific relation of facial trustworthiness with likability or relations with any valenced dimension of facial characteristics. This question is of theoretical importance because although evaluations of facial trustworthiness and attractiveness are correlated, they have been proposed to reflect unique core dimensions of face perception^65^. Exploratory analyses (see Supplemental Material on OSF) revealed similar results using facial attractiveness norms instead of facial trustworthiness norms^42^. These findings suggest the described patterns may reflect a more general effect of valenced facial characteristics on evaluated likability than a specific facial trustworthiness effect.

The present work adds to a growing body of work showing that affective polarization is reflected in basic aspects of person perception that affect countless parts of social cognition and interaction^17,18,66^. A strengthened relation between facial trustworthiness and evaluated likability among shared relative to opposing partisans suggests that partisanship effects on core dimensions of face perception may contribute to partisan tension. By identifying these effects, we highlight an important role of basic dimensions of face perception in this tension that may be critical to consider when developing interventions to reduce it.

## Supplementary Information

Supplementary Information.

## Data Availability

The datasets generated and analysed during the current study are availability in the Open Science Framework repository (https://osf.io/xdf6m/?view_only=a97a8ee15b044bcfbc0cbe7715ff610a).
