# Supplementary material for: Perceiver and target partisanship shift facial trustworthiness effects on likability
Source: Sci Rep. 2023 Apr 15;13:6130. doi: 10.1038/s41598-023-33307-8 (PMC10105733; doi:10.1038/s41598-023-33307-8)
Supplement: Supplementary file 1 — Supplementary Information. [file 41598_2023_33307_MOESM1_ESM.docx]

**Supplemental Material**

Experiment 1 linear mixed effects model substituting facial attractiveness (standardized) for facial trustworthiness (standardized)

|  | **Evaluated Likability** | | |
| --- | --- | --- | --- |
| *Predictors* | *Estimates* | *CI* | *p* |
| (Intercept) | 3.57 | 3.39 – 3.74 | <0.001 |
| Party Label [unlabeled] | -0.10 | -0.19 – -0.02 | 0.015 |
| Party Label [Republican] | -0.26 | -0.41 – -0.12 | 0.001 |
| Political Ideology | 0.18 | 0.01 – 0.35 | 0.033 |
| Attractiveness | 0.36 | 0.28 – 0.45 | <0.001 |
| Party Label [unlabeled] × Political Ideology | -0.24 | -0.32 – -0.15 | <0.001 |
| Party Label [Republican] × Political Ideology | -0.49 | -0.64 – -0.34 | <0.001 |
| Party Label [unlabeled] × Attractiveness | -0.02 | -0.06 – 0.03 | 0.476 |
| Party Label [Republican] × Attractiveness | -0.04 | -0.08 – 0.01 | 0.130 |
| Political Ideology × Attractiveness | 0.02 | -0.03 – 0.07 | 0.410 |
| (Party Label [unlabeled] × Political Ideology) × Attractiveness | -0.09 | -0.13 – -0.04 | <0.001 |
| (Party Label [Republican] × Political Ideology) × Attractiveness | -0.08 | -0.13 – -0.03 | 0.001 |

The strength and direction of the coefficients largely paralleled the model in the main text using facial trustworthiness. Thus, the facial trustworthiness effects described in the main text may reflect a more general effect of positive facial characteristics on evaluated likability than a specific facial trustworthiness effect. Future research may test this possibility more directly given that the most attractive faces are not necessarily the most trustworthy (e.g., Sofer et al., 2015).
